# Dry Needling of the Popliteus Muscle Validation by Ultrasound Imaging: A Cross-Sectional Observational Study

**DOI:** 10.3390/jcm11216409

**Published:** 2022-10-29

**Authors:** José-Ignacio De-Arriba-Agre, Carmen García-Mulas, Sara Grigelmo-Hernández, Jose-Jesús Jiménez-Rejano, Samuel Fernández-Carnero, Fermin Naranjo-Cinto, Daniel Pecos-Martín, Susana Nunez-Nagy

**Affiliations:** 1 Universidad de Alcalá, Facultad de Enfermería y Fisioterapia, Departamento de Fisioterapia, Grupo de 6 Investigación en Fisioterapia y Dolor, 28801 Alcalá de Alcalá, Spain; 2 Department of Physiotherapy, University of Seville, 41009 Seville, Spain

**Keywords:** dry puncture, popliteal muscle, ultrasound

## Abstract

Dry needling is a widely used technique for the treatment of painful syndromes in the musculature, however, its usefulness is of greater relevance in deep structures, such as the popliteus muscle, as it is more difficult to access. This muscle is heavily involved in knee pathology, being a source of pain and functional impairment, especially secondary to underlying pathologies. The method selected for the observation and study of the soft tissues, by means of imaging tests that do not use ionising radiation, is ultrasound. A cross-sectional observational study is proposed. It will be carried out in a healthy population, during the years 2021 and 2022, observing, by ultrasound, the results of the popliteal puncture technique, recorded by Mayoral del Moral et al. A popliteus muscle needle reach of 92% was achieved with this technique, in 48 of 50 patients. The results of the present cross-sectional observational study in living subjects, support that the popliteal puncture, described by Mayoral et al. is a reliable and safe approach, when performed with a 0.30 × 50 mm needle, and no adverse reactions or punctures of the vascular-nerve structures have been reported during the interventions.

## 1. Introduction

Dry needling (DN) is a technique used in physiotherapy to improve the patient’s myofascial symptomatology, affecting both the muscle and the connective tissues that surround it [1,2] It consists of the introduction of a small-gauge needle into a point of pain, passing through the skin and reaching the musculature, thus it is considered an invasive technique [1]. The name “dry” refers to the translation from English (dry needling) and to the non-introduction of medication into the patient’s body [2,3]. Various forms of application are described: depending on the tissue to be reached, it can be superficial or deep, or, depending on the way the needle is moved, there is the Hong technique (quick in and out) or the Baldry technique (static needle placement on the point for 30 s) [3].

There is extensive scientific literature on the efficacy of the DN technique, more specifically the deep technique, in the management of myofascial pain and pain of a muscular origin [4], although it is not clearly superior to other non-invasive techniques [5]. However, unlike other non-invasive techniques, BP allows more direct access to deep tissues and musculature.

Nevertheless, as an invasive technique, there are some possible adverse effects to take into consideration, such as the possibility of bleeding, bruise, pain during or after puncture, bending of the needle, blockage or breakage of the needle. Pneumothorax and vegetative reactions in patients have been reported as more serious adverse effects [3,6,7].

This technique has absolute contraindications, such as belenophobia and/or patients who are reluctant to perform the technique, due to a history of fear of an adverse reaction to needles, if the limb where the puncture technique is performed is an area with lymphoedema, and if informed consent is not obtained, due to difficulties in communication, comprehension or related to the age of the subject [6].

Furthermore, relative contraindications are alterations in the immune system (cancer, hepatitis, HIV, immunosuppressive treatments), debilitated patients or chronic diseases, rheumatoid arthritis, coagulation disorders (people undergoing anticoagulant therapy or taking drugs with anticoagulant effect, vascular pathologies), diabetes, pregnancy, patients who have difficulty communicating their sensations adequately, epilepsy, metal allergies, area with erosions or wounds, children under 13 years of age, prosthetic implants, electrical devices or in the case of malignant tumours or in their vicinity [3,6].

The anatomy of the popliteus has been the subject of controversy in recent years, with recent research discovering different anatomical variants relevant to establishing its function [8]. To date, it is known to be one of the few muscles in the body with an inverse origin and insertion, that is, the orientation of the fibres is oblique with respect to the flexion-extension axis of movement [8]. Classical descriptions speak of a triangular shape, an origin in the lateral condyle of the femur, capsule and posterior horn of the lateral meniscus, and an insertion in the postero-medial aspect of the tibia. Recent cadaveric studies show a high prevalence of insertion also in the head of the fibula, which some authors call the peroneal popliteal ligament [9].

The popliteal nerve is innervated by the tibial nerve (L5–S1) and supplied by the medial inferior genicular branch of the popliteal artery and the muscular branch of the posterior tibial artery. Lymphatic drainage extends to the popliteal nodes and subsequently drains to the deep inguinal nodes of the thigh [10].

The function of the popliteus is primarily stabilising, specifically of the postero-lateral compartment of the knee. It performs an internal rotation of the tibia, relative to the femur when the lower limb is unloaded, and an external rotation of the femur when the limb is loaded, which some authors describe as the “key”that unlocks the knee [8]. It therefore plays a key role in stabilising the posterolateral compartment of the knee [8,9], mainly in a static phase, under stress stimuli in an external rotation, knee varus and tibial translation. A. Wood et. al. also argue for an important involvement of the popliteus in the dynamic phase [11]. 

### Clinical Significance of the Popliteus Muscle

The clinical impact of the popliteus is poorly studied. It is known that its pathology is mostly secondary to injuries to the anterior and/or posterior cruciate ligament, the external meniscus or the external lateral ligament, and although some very particular cases of isolated tendinopathies have been described, injury to the muscle portion is much more frequent. Direct blows to the antero-medial aspect of the tibia, external rotation with the hyperextension of the knee or tibial fractures are some situations that can cause popliteal pathology [8], as well as downhill running situations with very long strides [12].

Regarding the pain it produces, Mayoral del Moral et al. [3] describe a pattern of referred pain in the region of the popliteal fossa and, less frequently, in the upper part of the gastrocnemius or even the antero-internal region of the knee. Ultrasonography has proven to be reliable, valid, economical, and direct for the assessment of this muscle [13]. For diagnosis, no specific and sensitive orthopaedic tests are described, therefore, apart from a clinical diagnosis, imaging tests are used; ultrasound and MRI have been described as the most useful forms of diagnosis [14,15,16]. Magnetic resonance imaging has been described as the most useful form of diagnosis [14,15,16].

Regarding invasive access to the popliteus and its risks, some cadaveric studies have described the possibility of injury to the vascular or nervous structures, during the application of infiltrations at the level of the popliteal fossa; however, other authors, such as Kampitak et al**.**, describe a safe way of infiltrating anesthetics at the level of the popliteal fossa in an ultrasound-guided manner, avoiding reaching vascular-nerve structures [14,15]. Regarding dry needling, there are no studies that validate this technique using ultrasound, which would have a major clinical impact. 

The popliteus is part of the deep musculature of the popliteal fossa, and therefore cannot be accessed by manual therapy for palpation and treatment, due to the superficial muscular structures and the vascular-nerve bundle. The most accessible treatment method is the invasive dry needling technique.

Currently, there is no evidence to prove that the dry needling technique is valid and reliable, and that it actually accesses the muscle. With the help of ultrasound, the soft tissue is clearly imaged, using a non-ionising medium for the patient.

The main objective of the present study is to determine if the dry needling technique, described by Mayoral et.al, really reaches the popliteus muscle using ultrasound imagery. 

## 2. Materials and Methods

### 2.1. Design

This is a cross-sectional observational study. It will be carried out in a healthy population, during a specific period of time, without prolonging it, observing the results of a technique to be performed on these patients. The STROBE (Strengthening Reporting of Observational Studies in Epidemiology) statement guidelines [17] have been followed for this study. The study was approved by the Ethics Committee of University of Alcalá (with approval number 2021/6/137). 

It was conducted by three qualified physiotherapists in Salamanca, at the ASyCO medical centre and the University of Alcalá, between 2021 and 2022. Initially, volunteers were recruited through social media, and with an informative poster placed in the university. Then, they were provided with the necessary information and informed consent was obtained. Subsequently, an intervention was carried out. 

Two experienced physiotherapists, both with the same dominant hand, performed the puncture on the asymptomatic volunteers. The lower limbs to be punctured were chosen at random, and each therapist performed two punctures. Subsequently, the physiotherapist who performed the puncture left the room and an ultrasound measurement was taken, to locate the point where the needle was located. The process was then be repeated with the second physiotherapist to assess whether or not both coincided, and if the puncture had indeed reached the popliteus. Therefore, two measurements were obtained for each patient. The same needle size, as described below, was be used for all patients. 

**Figure 1:**
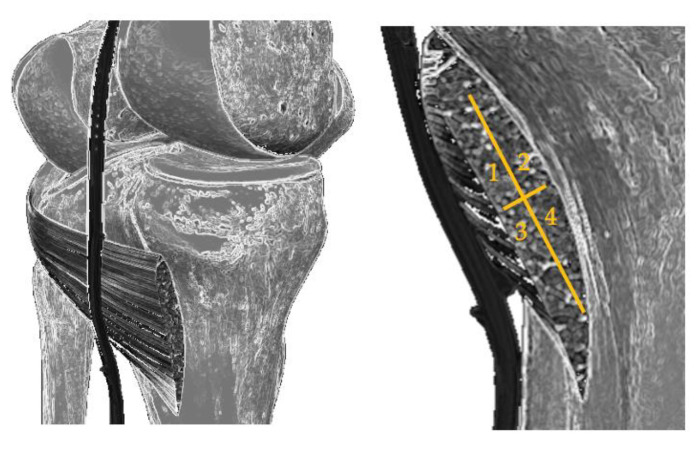
Diagram of the division of the popliteus into four quadrants.

For the analysis of the most reached region of the muscle and its possible relationship with the perceived pain by Verbal Numerical Scale (VNS), a diagram of the popliteus was made and was divided into four quadrants: internal superior (most medial side, closest to the point of access with the needle), external superior (most lateral side), internal inferior and external inferior (Figure 1).

### 2.2. Sample Size Calculation

The sample size calculation was performed with Epidat 4.2 software and was based on the precision of the proportion estimation. Assuming a popliteus muscle hit ratio of over 90%, a precision of 8.5%, an infinite population size and α error level of 0.05, the resulting sample size was 48 subjects. The final sample size was set at 50 subjects.

### 2.3. Population

The study was carried out on 50 volunteers, 28 healthy men and 22 healthy women, aged between 18 and 60 years. The demographic data (sex, age, weight and height) and the study variables: puncture of other structures (yes/no), presence of local twitch response (LTR) (yes/no), needle depth and numerical verbal scale were obtained for each of these volunteers. Thus, two dependent variables are obtained for each volunteer, multiplied by two interventions, in 50 volunteers. In addition, the subjects were asked if they had any sensation different from the previous ones, or if there was anything relevant to record. This resulted in a total of 200 dependent variables, i.e.**,** 100 measurements of each variable.

### 2.4. Inclusion Criteria

Healthy patients, with no previous pathology or symptomatology in the knee. They were between 18 and 60 years of age.

### 2.5. Exclusion Criteria

Patients with previous knee pathology, knee surgery or knee symptomatology within one year, pregnancy, belonephobia (fear of needles), patients with contraindications for dry needling, previous adverse reactions to needles, treatment with anticoagulants or antiplatelet agents, immune system disorders, infectious diseases, epileptic or neoplastic problems, puncture sites with lymphoedema, skin diseases (psoriasis, infections, etc.), wounds, erosions or scars and allergic to metals (especially nickel).

### 2.6. Method of Analysis

The statistical package SPSS version 20.0 for Windows was used for the statistical analysis. The normality was assumed for the continuous quantitative variables, according to the central limit theorem because the sample size was greater than 30 subjects. For the quantitative variables, the arithmetic mean and standard deviation (SD) were calculated and accompanied by a box-and-whisker plot for the most relevant variables, while for the qualitative variables, the absolute frequency and percentage of each category were shown, accompanied by a pie chart for the “punctured muscle region” and “other punctured structures”. For the analysis of concordance in the popliteus muscle puncture, the percentage of correct answers was calculated for each assessor.

The difference between physiotherapist 1 and 2 in the variables needle depth, VNS and LTR was also determined using Student’s *t*-test for the related samples. The box and whisker plots of this comparison are also shown.

Finally, the values for the punctured muscle regions and the puncture of other structures were also compared using McNemar’s test. Statistical tests were performed considering a 95% confidence interval (hereafter CI) and a significance level α = 0.05 (*p* < 0.05) [18].

### 2.7. Procedure

Two experienced physiotherapists performed the dry needling of the popliteus, both with the same dominant hand. The side to be pricked was chosen at random. Once the puncture was completed (explained in the following section) the therapist (hereafter referred to as physiotherapist 1) left the room to avoid bias, while the sonographer proceeded to check the needle location and measure the depth of the needle. The needle was then removed from the volunteer and, after a few minutes of rest, another therapist (hereafter referred to as physiotherapist 2) performed the second puncture, and the process was repeated in the same way. 

Therefore, two depth measurements and two hit measurements (YES/NO) were obtained from each volunteer. The same needle size, described below, was used for all patients. Regarding the variables studied, the patient was asked about the pain experienced during the whole process with the EVN [19], as well as other sensations that the subject considered relevant (e.g.**,** pain referred to other areas). The presence or absence of LTR when introducing the needle and whether the puncture of other important structures, other than the popliteus muscle (artery, vein, nerve) had been evidenced, were also considered.

### 2.8. Intervention

Prior to the puncture, the posterior knee area was disinfected with chlorhexidine and left for one minute. In the meantime, the physiotherapist put on gloves and prepared the equipment: needle, absorbent cotton wool and biohazard waste container. The patient was placed in the supine position, with the hip in slight abduction and external rotation, and the knee flexed at about 90°. A wedge or cushion was placed under the knee to be punctured to facilitate access, leaving the upper leg free, where the ultrasound machine was placed. The contralateral lower limb was extended and relaxed. The physiotherapist was positioned homolateral to the patient. 

Palpation of the muscle was performed at its insertion in the proximal part of the tibia, on the inner side. Physiotherapist was required to palpate the inner face of the tibia in its proximal third, two fingers measurement below the medial condile of femur, and tried to avoid the gastrocnemius muscles. 

The process was repeated until the needle was inserted about 30 mm without reaching the tibia. Here we reached the popliteus muscle [2,8].

The risk of puncture in the popliteal region is to reach the vascular nerve bundle. To avoid this, the needle had to be as close as possible to the posterior aspect of the tibia, always using the bone as a reference. Some superficial branches may have passed through the needle insertion area, so it was important to make a slow entry and assess the patient’s sensations [8].

**Figure 2:**
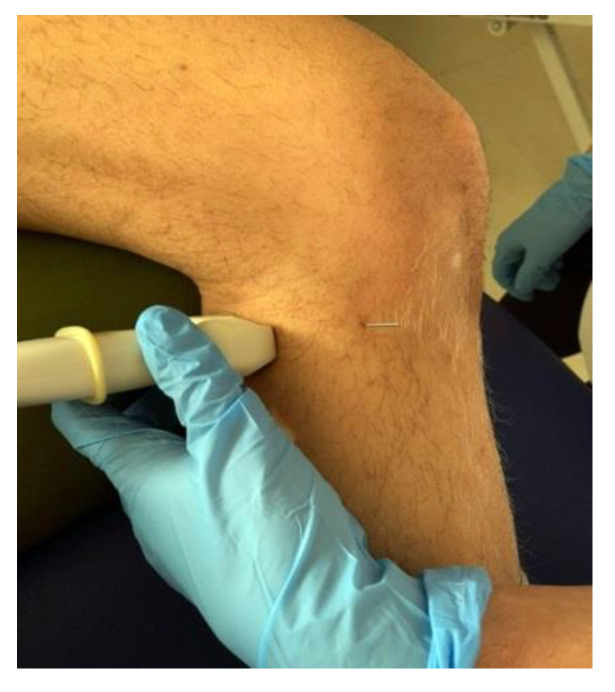
Position of the probe in relation to the needle.

For the ultrasound examination, a linear probe was used and a transverse plane was made, placing the notch of the probe to the patient’s right, as shown in Figure 2.

**Figure 3:**
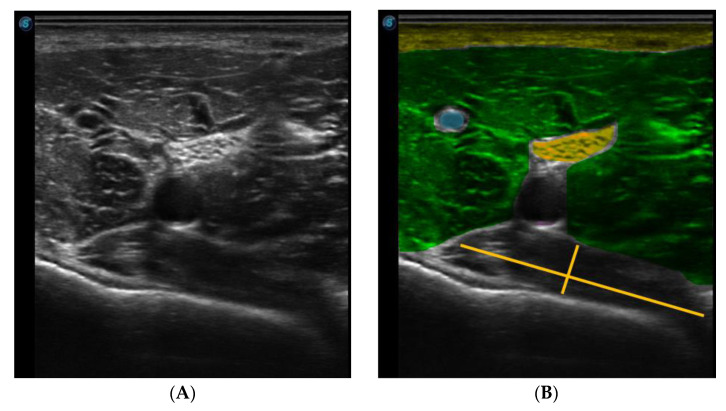
Ultrasound image of the popliteal region. (**A**) Ultrasound B-mode image (**B**) Colored Ultrasound B-mode image (rose-skin, yellow-fat, green-medialis gastrocnemius, blue-vein, magenta-artery, orange-nerve and red-popliteal muscle).

The needle was observed in a long axis, so that the entire length of the needle could be seen in the image. Figure 3 shows the ultrasound image of the popliteus muscle, in the deepest part, just below the vasculonervous bundle.

## 3. Results

The present study is a cross-sectional observational study, in which the variables described below were collected and analysed. The statistical package SPSS version 20.0 for Windows was used to analyse the data. 

### 3.1. Description of the Sample

The final sample size was 50 subjects (*n* = 50) aged between 18 and 56 years (SD = 24 ± 8.49 years), including 22 females and 28 males. 

All of the interventions were performed on the right upper limb, by the two therapists, while the lower limb to be punctured was chosen randomly, resulting in 50% right knees and 50% left knees. The weight (kg) and height (cm) data were collected for all subjects for the calculation of the Body Mass Index (BMI). The mean BMI value obtained was 22.9 0 ± 2.41, which is considered a normal weight. The values are explained in detail in Table 1. 

### 3.2. Analysis of the Quantitative Variables

The mean needle depth in all punctures was 4.60 cm (SD = 4.60 ± 1.60 cm), with a maximum depth of 4.98 cm (found in only one subject), and a minimum depth of 3.60 cm (without taking into account the 0 cm values that correspond to subjects in whom the popliteus was not reached). 

**Figure 4:**
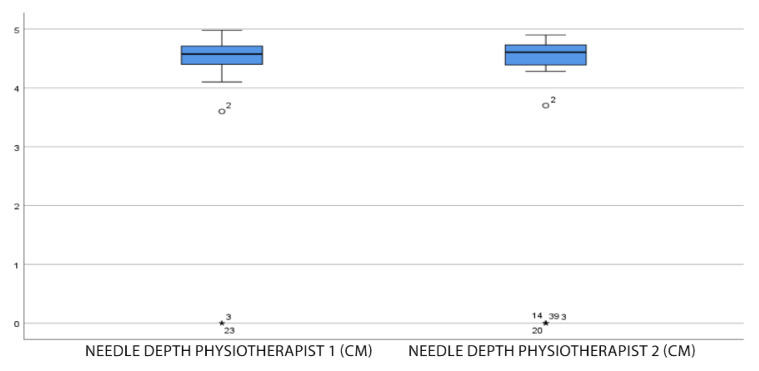
Box-and-whisker plot of the needle depth. The (*) outliers > 3 × IQR interquartile range.

As can be seen in the box-and-whisker plot (Figure 4), the majority of the punctures are between 4 and 5 cm, regardless of the therapist performing the puncture. The values outside the boxes correspond to the minimum values mentioned above (3.60 cm for physiotherapist 1 and 3.70 cm for physiotherapist 2).

**Figure 5:**
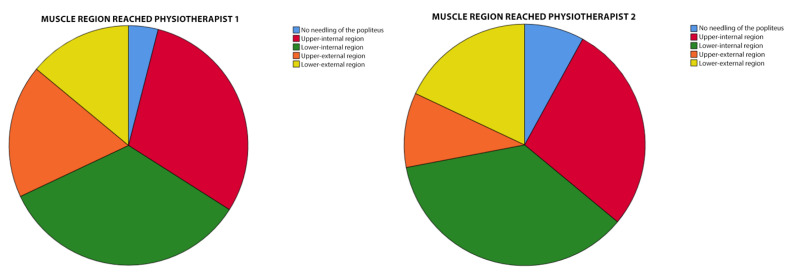
Sector diagram of the regions dotted by each therapist.

Regarding the area of the muscle punctured, it was found that, in the majority of the interventions, it was the internal inferior region of the muscle (34%) that was reached the most often, which corresponds to the most medial side and closest to the access point. This was followed by the upper inner region (30%), the upper outer region (18%) and finally, the least punctured region was the lower outer region (14%). These values correspond to the total number of subjects. The following pie charts show the division by region for each therapist (Figure 5).

**Figure 6:**
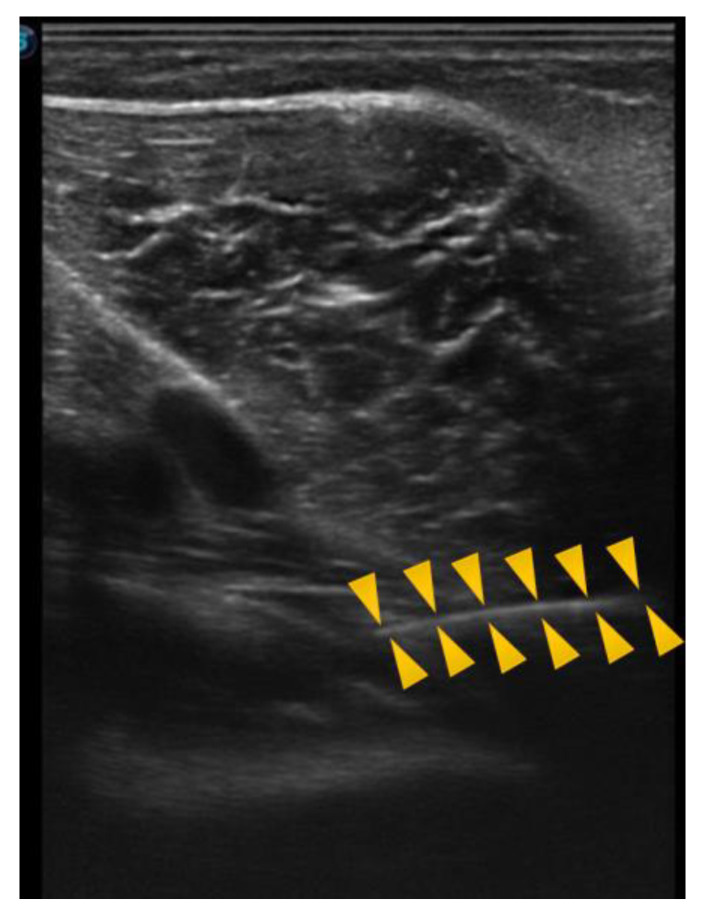
Ultrasound of the popliteal puncture in the internal superior region. Head arrows define the needle line.

In the following image, obtained directly from the ultrasound scanner, we can see an example of a puncture, in which the needle, entering from the right side of the screen, directly reaches the popliteus in the internal superior region, which was the most frequently punctured region (Figure 6).

To analyse the relationship between the variables of the BMI and the depth of the needle, a bivariate correlation analysis was performed, finding that there is no statistically significant relationship between these variables (Table 2).

The data relating to the local twitch response (LTR) indicate the number of patients in whom this spasm appeared, as it was treated as a categorical variable (YES/NO), finding an average of 18.5 spasms in the intervention of the first therapist, and 20.52 for the second therapist.

In relation to the popliteus muscle hit, in physiotherapists 1 and 2, there was total agreement in the hits in 46 patients. In only four patients, there was no popliteus muscle hit, of which in one there was agreement between the two physiotherapists on the lack of a hit. In the other three patients, either physiotherapist 1 or 2 was unable to hit the target. This shows a hit rate for the two physiotherapists on the popliteus muscle of 92% and a miss rate of 8% (Table 3).

In terms of variables related to the intervention, pain during the puncture was calculated using the numerical verbal scale. The mean value for physiotherapist 1 was 3.88/10 and for therapist 2, it was 3.76/10. A correlation was made between the patient’s VNS and spasm response, but no statistically significant relationship was found in any case (Table 4).

**Figure 7:**
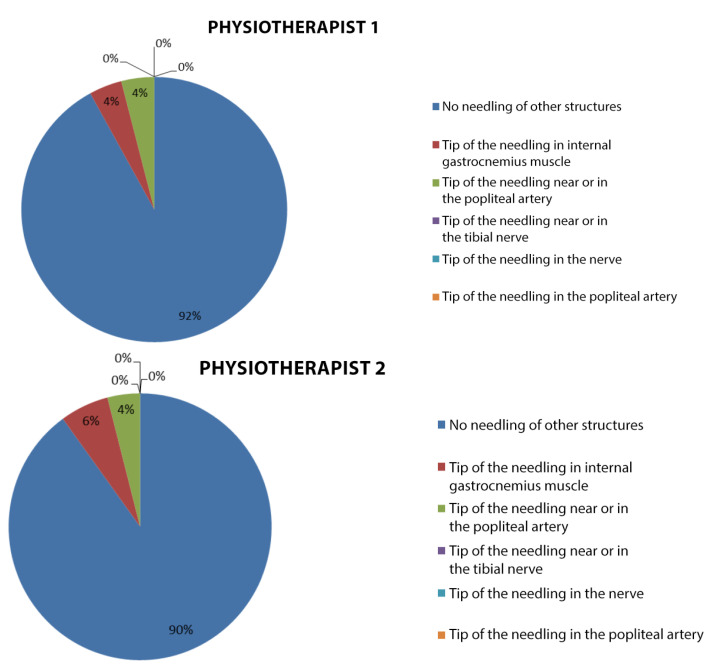
Sector diagram of each therapist’s punctures of other structures.

In the study, there was a possibility of puncturing other structures, yet only the gastrocnemius internus was punctured, other than the popliteus muscle. However, it was possible to observe in some patients that the needle passed close to other structures. The percentage of the needle proximity to the popliteal artery of 4%, was recorded by both physiotherapist 1 and physiotherapist 2, a proximity to the tibial nerve of 0% and the gastrocnemius puncture without a popliteal puncture amounted to 4% in physiotherapist 1 and 6% in physiotherapist 2. The percentage of both the popliteal artery and tibial nerve puncture was 0% in both physiotherapists. These data are detailed in Figure 7.

**Figure 8:**
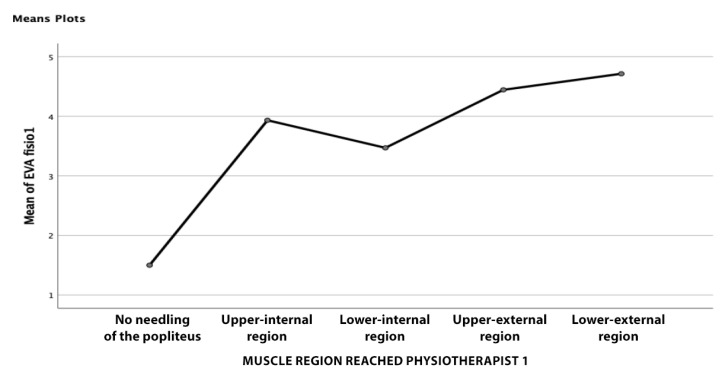
Line graph of the relationship between the VNS and punctured muscle region physiotherapist 1.

**Figure 9:**
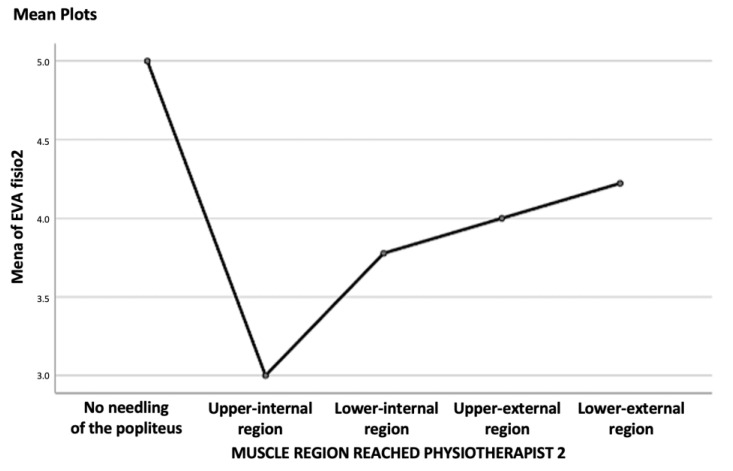
Line graph of the relationship between the VNS and the region of the punctured muscle for physiotherapist 2.

Finally, the possible relationship between pain (VNS) and the muscle region reached was studied by calculating a one-factor ANOVA. No statistically significant relationships were found for either therapist (*p* = 0.023 for therapist 1; *p* = 0.473 for therapist 2). The results, regarding this correlation are detailed in Table 5 and Table 6 and Figure 8 and Figure 9.

## 4. Discussion

To our knowledge, this is the first study to validate the safety and reliability of the dry puncture of the popliteus by ultrasound in living subjects. The results of the study show a high percentage of success in the popliteus (92%), using the puncture described by Mayoral O. et al. [3], being a safe approach, as no vascular-nerve structures were punctured. 

A needle size of 0.30 × 50 mm was found to be an optimal size, as in no case is the needle insufficient, taking into account the 5 mm safety margin that must be left outside of the skin. We only observed one subject in whom a longer needle would have been safer (a depth of 4.98 cm was reached); coinciding with a higher than average BMI. Although there was no significant relationship between the variables of the BMI and the needle depth, a direct relationship was observed, as the *p*-value was positive. This implies that the clinician should consider, in patients with larger anthropometric measurements, using a longer needle to ensure that the popliteus is reached and that a sweep of the needle can be made during entry and exit, but this is not common. 

An interesting finding was an increase in the perceived pain in subjects in whom the needle passed close to the popliteal artery, without reaching it directly. There is not a significant number of cases to establish a statistically significant relationship between the two events, but it is interesting for the clinician to consider that a possible increase in pain may correspond to being close to this structure. The only structure other than the popliteus that was punctured in 4% and 6% of the subjects, was the internal gastrocnemius, which did not pose a risk to the patient. 

Regarding pain, it’s known that it is one of the frequent limitations of dry needling. Pain has been related, in clinical practice, to the presence of LTR, although there is no evidence to support this [20]. In our study, we observed that there is no direct relationship between these variables, which coincides with previous research, which also suggests that it may not be necessary to provoke LTR during the puncture to improve the patient’s pain. 

This study also assessed whether the region of the muscle reached was relevant, in terms of perceived pain, and found that there was no relationship between the two variables. This implies that, during the puncture, there should be no significant change in pain, as the different regions are traversed. Furthermore, it implies that once the clinician considers that the muscle has been reached, there is no need for a change in the direction of the needle or an attempt to reach a particular region, as this will not result in a significant change in pain for the patient. 

In view of the above, we consider that the objectives set out in the study have been met and that they are relevant to clinical practice for several reasons; firstly, because of the important role of the popliteus in the biomechanics of the knee [9,12,13], which means that a change in its function, whether due to myofascial pain or another injury, can lead to a significant limitation in the joint and, consequently, in the patient’s functionality. Rodríguez-Sanz et al**. [21]**. set out similar objectives to the present study, managing to demonstrate that the puncture is safe and reliable when reaching the popliteus in nine out of 10 cadavers. Their results, therefore, are complementary to ours. 

Demonstrating the validity and safety of a technique that allows it to be approached directly, opens up the range of techniques and treatment possibilities, as well as giving the clinician peace of mind when performing the puncture. 

Ultrasound was chosen as the validation method because it does not present ionising radiation for the subject and because of its high reliability in visualising muscular and vascular-nerve structures, and because it allows invasive techniques to be performed at the same time as the ultrasound [13,15]. Its use has been used in recent years to study deep muscle puncture techniques, as can be seen in the work of Albin SR et al. [22], Bagcier F et al. [23] and Wang-Prince S et al. [24]. Albin et al. [22] performed a methodology very similar to this work on the tibialis posterior muscle. The puncture of the tibialis posterior is similar to that of the popliteus, in that it requires moving the needle very close to the tibia until it is no longer touching bone. In this case, they found that the optimal needle size was also 0.30 × 50 mm, nor were punctures of nearby vascular-nerve structures reported. They also described a specific needle orientation: the tip should be positioned towards the most ventral part of the patient and as close to the bone as possible, away from the midline, and the needle should be introduced gently and progressively. This coincides with the results of our study which, moreover, provides data from a sample size notably larger than that of the aforementioned studies. 

Other authors, such as Bagcier F. et al**.** [23], performed the validation in an ultrasound-guided manner, i.e., the muscle is punctured while the ultrasound is being performed, in order to ensure that the vascular-nerve structures are avoided. In our case, and in that of Albin et al. [22] and Wang-Prince S et al**.** [24], ultrasound is performed afterwards, and it has been shown that an ultrasound-guided puncture is not necessary, which is an enormous advantage for physiotherapists who do not have an ultrasound scanner, as it is still a resource that is not very accessible in the clinic. Comparing our study with previous studies mentioned, this trial is made on a greater sample size, which provides quality to the results. 

It is important to continue to provide evidence regarding the safety of dry needling in clinical practice, as its efficacy in pain management has been amply demonstrated [2,3,4,5]. However, the use of needling has been changing in recent years, calling into question aspects, such as local spasm responses, how many punctures are necessary, or even whether trigger points should really be the target of needling [25]. There is certainly merit in further research on this and on how to reassure the physiotherapist when performing invasive treatments. 

As take-home messages, we would emphasize the following points: clinicians should follow the instructions of Mayoral et al., for the needling of the popliteus, 0.30 × 0.50 mm is the correct needle size, pain should not change when going through different muscle regions, and finally, it is not necessary to have an eco-guide for this intervention. 

With regard to the limitations of our study, the distance between the needle tip and the vascular-nerve structures was not measured, which would be interesting to assess how much of a margin of distance is involved in order to minimize risk. Another limitation is that the intervention is made in healthy subjects, and it would be engaging its validation in patients with knee pain. 

## 5. Conclusions

The results of the present cross-sectional observational study in living subjects support that the popliteal puncture described by Mayoral et al. is a reliable and safe approach when performed with a 0.30 × 50 mm needle. No adverse reactions or punctures of vascular-nerve structures have been reported during the interventions. The clinical relevance of the popliteus necessitates further research into its treatment, and in particular into invasive techniques.

## Figures and Tables

**Table 1:** Descriptive analysis of the dependent and independent variables.

Variable	Mean	Standard Deviation	Maximum	Minimum
Age	26.82	8.49	56	18
Weight	68.92	11.67	92	40
Height	171.84	9.31	191	154
BMI	23.18	2.41	28.01	16.02
Needle depth 1	4.37	0.93	4.98	0
Needle depth 2	4.22	1.27	4.9	0
VNS 1	3.88	2.07	8	0
VNS 2	3.76	1.77	7	0
Local twitch response 1	18.5	0.505	-	-
Local twitch response 2	20.52	0.505	-	-

**Table 2:** Correlation between the BMI and the needle depth.

		Needle Depth Physiotherapist 1	Needle Depth Physiotherapist 2
CMI	Pearson Correlation	0.168	0.030
	Sig (1-tailed)	0.112	0.417

**Table 3:** Percentage of success for each physiotherapist.

		Physiotherapist 2		
		Not Reached	Reached	Total
PHYSIOTHERAPIST 1	Not Reached	1	1	2
	Reached	3	45	48
TOTAL		4	46	50
% TOTAL		8%	92%	100%

**Table 4:** Correlation between the VNS and the local spasm response.

		Vns Physiotherapist 1	Vns Physiotherapist 2	Ltr Physiotherapist 1	Ltr Physiotherapist 2
Vns Physiotherapist 1	Pearson correlation	1	0.467	−0.199	−0.097
	Sig (2-tailed)		0.001	0.165	0.501
	N	50	50	50	50
Vns Physiotherapist 2	Pearson correlation	0.467 **	1	0.000	−0.199
	Sig (2-tailed)	0.001		1.000	0.166
	N	50	50	50	50
Ltr Physiotherapist 1	Pearson correlation	−0.199	0.000	1	0.240
	Sig (2-tailed)	0.165	1.000		0.093
	N	50	50	50	50
Ltr Physiotherapist 2	Pearson correlation	−0.097	−0.199	0.240	1
	Sig (2-tailed)	0.501	0.166	0.093	
	N	50	50	50	50

** significative < 0.01.

**Table 5:** Correlation data between the pain and muscle region for physiotherapist 1.

VNS				95% Confidence Interval for Mean				
	N	Mean	Std. Deviation	Std. Error	Lower Bound	Upper Bound	Minimum	Maximum
No needling of the popliteus	2	1.50	0.707	0.500	−4.85	7.85	1	2
Upper-internal region physiotherapist 1	15	3.93	2.052	0.530	2.80	5.07	1	7
Lower–internal region physiotherapist 1	17	3.47	2.239	0.543	2.32	4.62	0	8
Upper external region physiotherapist 1	9	4.44	1.667	0.556	3.16	5.73	2	7
Lower internal region physiotherapist 1	7	4.71	1.704	0.644	3.14	6.29	2	7
TOTAL	50	3.88	2.027	0.287	3.30	4.46	0	8

**Table 6:** Correlation data between the pain and muscle region for physiotherapist 2.

VNS					95% Confidence Interval for Mean			
	N	Mean	Std. Deviation	Std. Error	Lower Bound	Upper Bound	Minimum	Maximum
No needling of the popliteus	4	5.00	2.828	1.414	0.50	9.50	1	7
Upper-internal region physiotherapist 2	14	3.00	1.617	0.432	2.07	3.93	0	6
Lower-internal region physiotherapist 2	18	3.78	1.592	0.375	2.99	4.57	1	6
Upper external region physiotherapist 2	5	4.00	1.581	0.707	2.04	5.96	2	6
Lower external region physiotherapist 2	9	4.22	1.856	0.619	2.80	5.65	2	7
TOTAL	50	3.76	1.779	0.252	3.25	4.27	0	7
